# Preharvest Environmental and Management Drivers of Multidrug Resistance in Major Bacterial Zoonotic Pathogens in Pastured Poultry Flocks

**DOI:** 10.3390/microorganisms10091703

**Published:** 2022-08-24

**Authors:** Moses B. Ayoola, Nisha Pillai, Bindu Nanduri, Michael J. Rothrock, Mahalingam Ramkumar

**Affiliations:** 1 Geosystems Research Institute, Mississippi State University, Starkville, MS 39762, USA; 2 College of Veterinary Medicine, Mississippi State University, Starkville, MS 39762, USA; 3 Computer Science & Engineering, Mississippi State University, Starkville, MS 39762, USA; 4 Egg Safety and Quality Research Unit, USDA-ARS U.S. National Poultry Research Center, Athens, GA 30605, USA

**Keywords:** antimicrobial multidrug resistance, foodborne pathogens, food safety

## Abstract

Due to nutritional benefits and perceived humane ways of treating the animals, the demand for antibiotic-free pastured poultry chicken has continued to be steadily rise. Despite the non-usage of antibiotics in pastured poultry broiler production, antibiotic resistance (AR) is reported in zoonotic poultry pathogens. However, factors that drive multidrug resistance (MDR) in pastured poultry are not well understood. In this study, we used machine learning and deep learning approaches to predict farm management practices and physicochemical properties of feces and soil that drive MDR in zoonotic poultry pathogens. Antibiotic use in agroecosystems is known to contribute to resistance. Evaluation of the development of resistance in environments that are free of antibiotics such as the all-natural, antibiotic-free, pastured poultry production systems described here is critical to understand the background AR in the absence of any selection pressure, i.e., basal levels of resistance. We analyzed 1635 preharvest (feces and soil) samples collected from forty-two pastured poultry flocks and eleven farms in the Southeastern United States. CDC National Antimicrobial Resistance Monitoring System guidelines were used to determine antimicrobial/multidrug resistance profiles of *Salmonella*, *Listeria*, and *Campylobacter*. A combination of two traditional machine learning (RandomForest and XGBoost) and three deep learning (Multi-layer Perceptron, Generative Adversarial Network, and Auto-Encoder) approaches identified critical farm management practices and environmental variables that drive multidrug resistance in poultry pathogens in broiler production systems that represents background resistance. This study enumerates management practices that contribute to AR and makes recommendations to potentially mitigate multidrug resistance and the prevalence of *Salmonella* and *Listeria* in pastured poultry.

## 1. Introduction

The increasing incidence of antibiotic resistance (AR) is a major global threat to public health. Apart from the main usage of antibiotics for the treatment of infection, there is also prophylactic use to enhance growth in commercial poultry production in some countries. Antibiotics as growth promoters were banned in the EU in 2006 and in the US in 2017 [1]. The persistent presence of antibiotics in the environment could enhance the expression of antibiotic resistance genes and potentiate antibiotic-resistant bacteria [2,3]. In some scenarios, pathogens may develop resistance to multiple antibiotics/drugs, i.e., multidrug resistance (MDR) [4]. In addition to the overuse and misuse of antibiotics, co-selection of resistance genes due to the usage of biocides [5,6] or the presence of heavy metals [7] found naturally in the soil in agricultural environments can contribute to AR. The prevalence of MDR poultry pathogens such as *Salmonella* spp., *Listeria spp.*, and *Campylobacter spp.* is a food biosafety concern. Poultry is a major repository of these pathogens, and millions of people depend on poultry products for their daily protein supply. According to CDC, *Salmonella spp.*, *Listeria spp.*, and *Campylobacter spp.* are among the top nine causative agents of foodborne illnesses reported in the US [8].

The AR transmission mode and rates between animals, the environment, and humans is not well understood for all pathogens. As the potential risk of exacerbating AR in humans is a major concern, global efforts to mitigate AR are required. *Salmonella* MDR in pastured poultry is reported to be comparable to the MDR observed in conventional poultry production systems [9]. Specific farm management practices and/or physicochemical properties of the preharvest samples (feces, soil) that could contribute to the development of MDR are not known. Here, we sampled eleven different antibiotic-free pastured poultry farms, representing forty-two flocks in the Southeastern United States. *Salmonella*, *Campylobacter*, and *Listeria* were isolated from preharvest samples (feces and soil), and AR profiles were characterized utilizing National Antimicrobial Resistance Monitoring System for Enteric Bacteria (NARMS) protocols (www.cdc.gov/narms, accessed on 1 July 2021). To determine the management and environmental drivers of MDR among these pathogens, an ensemble machine learning approach comprising both traditional learning and deep learning was employed.

## 2. Materials and Methods

Forty-two flocks from eleven pastured poultry farms located in the southeastern U.S. were sampled over a period of four years. All broilers flocks were considered all-natural, pasture-raised, and never had any antibiotics administered to them during their grow-out, nor were they used historically on the farms.

**Sample Collection:** Preharvest samples (feces and soil) were collected from the pasture (i) within a few days of broilers being placed on the pasture, (ii) halfway through their time on pasture, and (iii) on the day the flock was processed. At each sampling time, the pasture area was divided into five separate sections, and five subsamples in each section were pooled into a single sample for each section (five total samples each of feces and soil samples were collected on each sampling day). The total volume of sample collected for each field sample was at least 25 g. All samples were collected in the field, and returned to the lab in a cooler packed in ice. Three grams (feces, soil) were combined within filtered stomacher bags (Seward Laboratories Systems, Inc., West cSussex, UK) and diluted 1:3 using 10 mmol/L phosphate-buffered saline (PBS). All samples were homogenized for 60 s, and homogenates were used for all downstream cultural isolations.

### 2.1. Cultural Isolation Methods

***Salmonella spp.:*** As a pre-enrichment step, the stomached homogenates remained in the filtered stomacher bags and were incubated overnight at 35 ${}^{\circ }$C. Two different enrichment broths were used to isolate *Salmonella spp.* from these environmental samples: Tetrathionate broth and Rappaport Vassiliadis (Becton Dickinson, Sparks, MD, USA) media. After overnight incubation at 42 ${}^{\circ }$C in both of these enrichment broths, one loopful from each enrichment broth was spread on two different differential media: Brilliant Green Sulfa with novobiocin (Becton Dickinson, Sparks, MD, USA) agar and xylose lysine tergitol-4 (Becton Dickinson, Sparks, MD, USA) agar. These plates were incubated overnight at 35 ${}^{\circ }$C, and on each plate, three *Salmonella*-like colonies per subsample were picked and confirmed using triple sugar iron agar (Becton Dickinson, Sparks, MD, USA) and lysine iron agar fermentation (Becton Dickinson, Sparks, MD, USA) using an incubation period of 18 to 24 h. Final confirmation of suspect triple sugar iron/lysine iron agar isolates was performed using *Salmonella* polyvalent O antiserum agglutination (Becton Dickinson, Sparks, MD, USA) using the manufacturer’s specifications. Positive *Salmonellae* were serogrouped using individual *Salmonella* poly O antisera for O groups A through I following the Kauffman–White scheme [10].

***Campylobacter spp.:*** Recovery of *Campylobacter spp.* from homogenized samples was performed as previously described [11]. Initially, 100 mL of homogenized suspension was removed, plated onto Campy–Cefex agar, and incubated at 42 ± 1 ${}^{\circ }$C for 36 to 48 h in a microaerobic atmosphere (5N2). Putative *Campylobacter spp.* colonies were enumerated, and up to five colonies per sample were subcultured on Brucella agar supplemented with 10% lysed horse blood (BAB plates) for isolation and incubated as previously described.

***Listeria spp.:*** As a pre-enrichment step, the stomached homogenates remained in the filtered stomacher bags and were incubated overnight at 35 ${}^{\circ }$C. This pre-enrichment step was followed by two enrichments in UVM-Modified *Listeria* Enrichment Broth (Becton Dickinson, Sparks, MD, USA) and Fraser Broth (Becton Dickinson, Sparks, MD, USA), both requiring overnight incubation at 30 ${}^{\circ }$C. One loopful of the Fraser’s enrichment was streaked for isolation of *Listeria* selective agar (Becton Dickinson, Sparks, MD, USA). These plates were incubated overnight at 30 ${}^{\circ }$C, and on each plate, three *Listeria*-like colonies per positive subsample were picked and confirmed as *Listeria* using the appropriate BAX PCR assay (DuPont).

### 2.2. Antibiotic Sensitivity Testing

For all three target bacteria, the published NARMS protocols and NARMS breakpoints were used for characterization and AR determination for each isolate (www.cdc.gov/narms, accessed on 1 July 2021). Isolates were considered multidrug resistant if they were resistant to three or more tested antibiotics.

***Salmonella spp.:*** Recovered isolates were subcultured on blood agar plates (BAPs) overnight at 36 ± 1 ${}^{\circ }$C, twice sequentially. One or two colonies were used to inoculate 5 mL of demineralized water to achieve a 0.5 McFarland equivalent using the Sensititre nephelometer (Thermo Fisher Scientific, Waltham, MA, USA). After vortexing, 10 mL of the cell suspension was transferred to 11 mL of Sensititre Cation adjusted Mueller–Hinton Broth with TES, followed by thorough vortexing. Fifty microliters of the inoculum was transferred to each well of the Sensititre NARMS Gram-Negative Format CMV3AGNF plate (Thermo Fisher Scientific, Waltham, MA, USA). These antibiotic sensitivity testing plates contained varying concentrations of the following antimicrobials in µg/mL: cefoxitin (0.5–32), azithromycin (0.12–16), chloramphenicol (2–32), tetracycline (4–32), ceftriaxone (0.25–64), amoxicillin/clavulanic acid (2:1) (1/0.5v32/16), ciprofloxacin (0.015–4), gentamicin (0.25–16), nalidixic acid (0.5–32), ceftiofur (0.12–8), sulfisoxazole (16–256), trimethoprim/sulfamethoxazole (0.12/2.4–4/76), ampicillin (1–32), and streptomycin (32–64). Plates were sealed and incubated at 36 ± 1 ${}^{\circ }$C for 24 h. Quality control strains *E. coli ATCC25922, Staphylococcus aureus ATCC 29213, Enterococcus fecalis ATCC 29212,* and *Pseudomonas aeruginosa ATCC 27853* were included in susceptibility tests as controls (Clinical and Laboratory Standards Institute, 2010).

***Campylobacter spp.:*** Recovered *Campylobacter spp.* isolates were subcultured on BAPs overnight at 42 ± 1 ${}^{\circ }$C. Three or four colonies were used to inoculate 5 mL of Sensititre Cation adjusted Mueller–Hinton Broth with TES (Thermo Fisher Scientific, Waltham, MA, USA) to achieve a 0.5 McFarland equivalent using the Sensititre nephelometer. After vortexing, 100 mL of the suspension was transferred to 11 mL of Sensititre Cation adjusted AutoRead Mueller–Hinton Broth with TES and 5% lysed horse blood. One hundred microliters of the inoculum was transferred to each well of the Sensititre CAMPY custom-made microtiter panel as previously described [12,13]. The panel contains nine antimicrobials with the following range of concentration in µg/mL: azithromycin (0.015–64), ciprofloxacin (0.015–64), clindamycin (0.03–16), erythromycin (0.03–64), florfenicol (0.03–64), gentamicin (0.12–32), nalidixic acid (4–64), telithromycin (0.015–8), and tetracycline (0.06–64). Panels were incubated under microaerobic conditions at 42 ± 1 ${}^{\circ }$C for 24 h, and *Campylobacter jejuni isolate 33560* [14] was used as a quality control organism.

***Listeria spp.:*** Recovered isolates were subcultured on BAPs overnight at 36 ± 1 ${}^{\circ }$C, twice sequentially. Colonies were used to inoculate 5 mL of Sensititre Cation adjusted Mueller–Hinton Broth with TES to achieve a 0.5 McFarland equivalent using the Sensititre nephelometer (Thermo Fisher Scientific, Waltham, MA, USA). After vortexing, 50 mL of the cell suspension was transferred to 11 mL of Sensititre Cation-Adjusted Mueller–Hinton Broth with TES with lysed horse blood, followed by thorough mixing by inversion. One hundred microliters of the inoculum was transferred to each well of the Sensititre NARMS Gram-Positive Format CMV3AGPF plate (Thermo Fisher Scientific, Waltham, MA, USA). These antibiotic sensitivity testing plates contained varying concentrations of the following antimicrobials in µg/mL: tigecycline (0.015–0.5), tetracycline (1–32), chloramphenicol (2–32), daptomycin (0.25–16), streptomycin (512–2048), tylosin tartrate (0.25–32), quinupristin/dalfopristin (0.5–32), linezolid (0.5–8), nitrofurantoin (2–64), penicillin (0.25–16), kanamycin (128–1024), erythromycin (0.25–8), ciprofloxacin (0.12–4), vancomycin (0.25–32), lincomycin (1–8), and gentamicin (128–1024). Plates were sealed and incubated at 36 ± 1 ${}^{\circ }$C for 24 h. A quality control strain (*Streptococcus pneumoniae* ATCC 49619) was included in susceptibility tests as a positive control (Clinical and Laboratory Standards Institute, 2010). Reported minimum inhibitory concentration values were divided by two to compensate for the double volume of inoculum transferred to plate wells, and the NARMS *Enterococcus* breakpoints were used for all drugs except penicillin, which has a *Listeria*-specific breakpoint listed.

### 2.3. Physicochemical Analysis

Feces and soil samples were analyzed as previously described [15]. The moisture content of the fecal and soil samples was determined by drying overnight at 65 ${}^{\circ }$C and calculating the difference between the wet and dried weights of the soil/feces. Fecal and soil pH and electrical conductivity (EC) were determined using an Orion Versa Star Advanced Electrochemistry Meter (Thermo Fisher Scientific, Waltham, MA, USA) using a 1:5 dilution in distilled water. Fecal and soil samples were submitted to the University of Georgia Soils Testing Laboratory (Athens, GA, USA) for determining the elemental composition.

Thirty-one distinct farm variables and management practice variables (Appendix A Table A1) associated with the feces and soil that were inputs for RandomForest algorithm [16] were used with an ensemble of five different machine learning approaches. Furthermore, the following twenty-four constituents/properties of poultry feces and pastured soil were included as additional input variables for models in this study: acidity/alkalinity (pH), electrical conductivity (EC), moisture, total carbon (TotalC), total nitrogen (TotalN), carbon-to-nitrogen ratio (C:N), aluminum (A), boron (B), calcium (Ca), cadmium (Cd), chromium (Cr), copper (Cu), iron (Fe), potassium (K), magnesium (Mg), manganese (Mn), molybdenum (Mo), sodium (Na), nickel (Ni), phosphorous (P), lead (Pb), sulfur (S), silicon (Si), and zinc (Zn).

### 2.4. MDR Prediction

An imbalanced, noisy, complex data set presents a major challenge in predicting MDR. This research uses predictive analytics to estimate the likelihood of MDR in *Salmonella*, *Campylobacter*, and *Listeria* by using historical poultry farm management variables. In our predictive modeling, we rely primarily on three processes: (1) standardization or normalization to reduce redundancy; (2) over-sampling to balance skewed distribution; and (3) deep learning to enhance confidence in MDR prediction (Please visit https://github.com/nispillai/EnsembleModellingForFoodSafety (accessed on 26 June 2022) for code). A pipeline combining quantile transformation, random sampling, and auto-encoding was created for the classification of MDR in *Salmonella*. Our auto-encoder model consists of an encoder layer and a decoder layer with 70 neural network units each and a latent layer with 30 neural network units. Additionally, we have a prediction model using 70 neural network units and a binary output node that can predict positive and negative samples from the latent representation. We use HE uniform [17] for the initialization of our kernel weights, Adam [18] for stochastic gradient descent, and ReLU [19] for activation. *Campylobacter* MDR is classified using unit normalization, SMOTE sampling, and multi-layer perception (MLP) framework. We used 30 neural network units in the hidden layer, along with a binary classification output layer. The weights of our kernels are initialized with HE uniform and Adam algorithms for stochastic gradient descent. In addition, we used robust-scale normalization, SMOTE sampling, and generative adversarial networks (GAN) to classify MDR in *Listeria*. For GAN, the discriminator model is composed of a hidden layer with 50 units and an output node. In the generator model, there are three layers: a hidden layer of 70 units, a latent layer of 50 units, and a generator layer of 70 units. We compute the results using the ReLU activation function and Adam optimizer, as well as HE uniform kernel initialization. We used four-fold cross-validation for all the models to train, evaluate, and predict the data sampled from the dataset. Our analyses are performed by using Python (v3.7.11), Tensor-flow (v1.15) [20], Keras (v2.3.1) [21], scikit-learn (v1.0.2) [22], pandas (v1.3.4) ([23,24]), and imblearn (v0.9.0) [25]. To perform the normalization, we used scikit-learn libraries with default parameters. We used the imblearn library with default parameters for oversampling approaches.

### 2.5. Critical Farm Feature Selection

In order to determine the critical and influential farm practices associated with MDR, we use a multi algorithm ensemble approach. In addition to the three deep learning algorithms (MLP, GAN, and Auto-Encoder), we also use popular machine learning algorithms such as Random Forest and XGBoost in this ensemble model. Following the training of the five learning models with the same data, we use the SHAP (SHapley Additive exPlanations) library (v0.39.0) [26] to select the top 15 most important features from all the models. We use majority voting, i.e., features selected by at least three models, to determine the most influential features. Once the influential features have been selected, we analyze the SHAP plots to determine which feature values are most likely to result in a reduction in MDR.

## 3. Results and Discussion

There is growing interest in antibiotic resistance (AR) and multidrug resistance (MDR) in the agricultural sector, as they are public health concerns especially for both clinicians and veterinarians. The use of antibiotics for the treatment of animal infections and prophylactic use at sub-therapeutic dose to enhance animal growth could lead to MDR and transmission to humans. About 148 mg of antibiotics per kilogram of chicken is estimated to be consumed by humans, and the quantity is projected to continue in an upward direction [27]. The application of animal waste as organic manure is an indirect environmental mechanism of AR transmission to humans as these anthropogenic contaminants could potentially enhance antibiotic resistance genes of the microorganisms in the ecosystem [28]. Nevertheless, microorganisms such as *Pseudomonas aeruginosa*, *Stenotrophomonas maltophilia*, and *Enterococci* possess intrinsic capability to develop antibiotic resistance without encountering any antimicrobial contaminant ([29,30]). Recent studies report the prevalence of MDR (resistance to three or more antibiotics) isolates of *Salmonella*, *Listeria*, and *Campylobacter*, important zoonotic pathogens on poultry farms without historical or exogenous sources of antibiotics [31]. However, the drivers of MDR from the environment (soil) or animals themselves (feces) are yet to be identified. We utilized an ensemble approach combining machine learning and deep learning to identify preharvest management practices that are predictive of MDR in poultry pathogens and determine the feature values that are associated with low MDR.

As stated in Section 2.4, our implementation uses some popular machine learning and data science libraries. We use Tensorflow, which is an open source library for implementing deep neural networks. In order to facilitate faster experimentation, we use Keras, a deep learning API that runs on top of Tensorflow. The scikit-learn package is another popular Python-based API that is used for machine learning algorithms. In addition, the pandas package is commonly used for data manipulation and analysis.

### 3.1. Data Analysis

Table 1 is an overview of the number of samples in our dataset that exhibit varying degrees of antibiotic resistance.

Only 27% of fecal samples had *Salmonella* that were resistant to three or more antibiotics (MDR), while 37% of *Salmonella* isolates were MDR in soil samples. *Salmonella* MDR included antibiotics that inhibit protein synthesis (tetracycline, streptomycin) and antibiotics that target cell wall synthesis (ampicillin, augmentin, cefoxitin, ceftriaxone, ceftiofur) among the antibiotics tested in this study. High prevalence (frequency of PCR-based detection) of the *tetA* and *aadA1* genes responsible for *E. coli* resistance to protein-synthesis-targeting tetracycline and streptomycin (72 and 33% prevalence rate, respectively) have been previously reported in poultry broiler chicken [32]. The same study found a similar pattern of resistance to protein-targeting antibiotics in layers. The horizontal transfer of resistance genes between *E. coli* and other organisms such as *Salmonella*, *Listeria*, and *Campylobacter* in the poultry production environment could contribute to the development of AR in these zoonotic poultry pathogens. *Salmonella* isolates resistant to a combination of tetracyline, streptomycin, and ampicillin, as observed in this study, have been reported in human, animal, and environmental samples [33]. Known mechanisms of resistance to these antibiotics involve bacterial plasmid and chromosomal DNA ([34,35]). Our results also show that 97.8% of the *Salmonella* isolates with MDR are of the Kentucky serotype and are of lesser public health concerns in humans, consistent with an earlier observation [9]. Only 1.1% of MDR *Salmonella* isolates belonging to the Braenderup/Cholareasuis strains are of significant importance to human health [36], while the remaining 1.1% represent ungrouped serotypes.

Our results show that only 5% of *Campylobacter* isolates were MDR in feces and soil. This dataset is highly imbalanced from the perspective of machine learning, making it difficult to build a generalized predictive model. The MDR *Campylobacter* identified in this study include *C. jejuni* (43%), *C. coli* (43%), and a mixed culture of *C. jejuni* and *C. coli* (14%). C_MDR included antibiotics that inhibit protein synthesis (azithromycin, gentamicin, clindamycin, erythromycin, florfenicol, telithromycin, tetracycline) and antibiotics that target DNA replication (ciprofloxacin and nalidixic acid). Similar to our observation, Aksomaitiene and colleagues reported *Campylobacter* resistance to tetracycline and ciprofloxacin in broiler chickens, as well as in humans, wild birds, and cattle [37]. Noreen et al. reported a high level of MDR in livestock-associated *Campylobacter* isolates compared to *Campylobacter* isolates from non-livestock sources such as water and wildlife [38]. Furthermore, their work identified *Camplobacter* MDR to antibiotics such as erythromycin, ciprofloxacin, nalidixic acid, tetracycline, and gentamicin, as observed in this study. Mutations in *tetO*, *aphA*, and *aadE* genes that could alter antibiotic binding sites, the presence of super efflux pump *RE-CmeABC*, and the carriage of *pTet* plasmid that could confer resistance could be the mechanisms for the development of *Campylobacter* MDR ([39,40]).

The MDRs observed in this study for *Listeria* in feces and soil samples are 72% and 85%, respectively. *Listeria* MDR predominantly involves antimicrobials that inhibit protein synthesis such as tetracycline, streptomycin, daptomycin, lincomycin, erythromycin, streptogramins, and tigecycline. Resistance to ciprofloxacin that inhibits DNA replication was also observed. MDR isolates of *Listeria* include *L. innocua* (78.3%), *L. welshimeri* (14.1%), *L. monocytogenes* (7.1%), and uncharacterized (0.5%). These results are consistent with the report of Okorie-Kanu and colleagues that identified *L. innocua* as the predominant species in chicken [41]. Although *L. innocua* is generally known to be non-pathogenic but genetically closely related to pathogenic *L. monocytogenes*, atypical hemolytic *L. innocua* has been reported to be virulent, albeit to a lower extent compared to *L. monocytogenes* [42]. A recent report indicates that *L. innocua* is capable of causing disease in farm animals [43]. Similar to the observation in this study, *Listeria* MDR to ciprofloxacin, tetracycline, and erythromycin has been previously reported in environmental samples [44]. Similar to *Salmonella* and *Campylobacter* MDR, mechanisms of *Listeria* MDR could involve both chromosomal and plasmid DNA [45].

MDR rates reported here corroborate the reported rates of 36.0%, 1.4%, and 63.9% in *Salmonella*, *Campylobacter*, and *Listeria*, respectively, in a survey of six pastured poultry farms [31].

### 3.2. Predictive Analysis

Our study examined preharvest feces and soil as separate models and a common model (combined soil and feces) for MDR classification in *Salmonella*, *Campylobacter*, and *Listeria*. These models aim to estimate the likelihood of pastured poultry farms developing MDR as a function of the farm management practices. Prior to building a prediction model based on the data, we standardize/normalize and oversample the data as described in Section 2.4.

**Standardization:** The goal of normalization is to convert numeric values in a dataset to a standardized scale while maintaining the differences in range. We compared the classification performance of four different normalization methods (unit normalization, robust-scale standardization, quantile transformation, and standard scale normalization) with our data. Our preliminary analysis showed that quantile transformations, unit normalization, and robust-scale normalization were effective in the classification of MDR in *Salmonella*, *Campylobacter*, and *Listeria*, respectively. **(a) Unit Normalization:** This method normalizes every sample, shrinking/stretching the input feature vector (x) to a unit sphere. This ensures that the vector scales to the unit norm without regard to the distribution of the samples (Equation (1)):

(1) $$X=\frac{x}{\left||x|\right|}$$

**(b) Robust-Scale Standardization:** This scaler centers and scales each feature independently using the quantile range (IQR: Interquartile Range) to reduce the influence of outliers in the feature set. Instead of considering the mean to standardize the feature, the method uses the median that is less significant to the outliers in scaling. **(c) Quantile Transformation:** A quantile function provides an approximation of the quantile positions of actual values by inversely calculating the cumulative distribution function. In quantile transformations [46], imbalanced distributions of data with outliers are converted to uniform distributions. This non-linear transformation smooths out the relation between observations by removing the linear correlation between the input variables. It is a popular and effective way to improve prediction with complex and noisy inputs. **(d) Standard-Scale Normalization:** Standardization is beneficial when the distribution of feature values follows a Gaussian distribution. It is a method for transforming feature values by subtracting from the mean and dividing by the standard deviation. Alternatively, this process is referred to as z-score standardization.

**Oversampling:** The percentage of negative samples outweighs the percentage of positive samples in our data. Oversampling is a technique often used to balance such skewed distributions. It maintains class balance by adding new points to the minority class rather than removing them from the majority class. We tested two popular oversampling strategies to balance the distribution during data processing. In our preliminary evaluations, random sampling proved effective for *Salmonella* MDR classification, while SMOTE sampling was effective for prediction of MDR in *Campylobacter* and *Listeria*. **(a) Random Oversampling:** In random oversampling [25], an increase in sample size is achieved by selecting minority class examples in random order and including them in the training set. This sampling technique iterates until a majority sample equals a minority sample. **(b) Synthetic Minority Oversampling Technique:** SMOTE [47] is based on the selection of a random sample from the minority class and one of its nearest neighbors, followed by the generation of a new synthetic sample within that range. Oversampling uses the nearest neighbor method in place of adding random duplicate samples to the minority class.

**Deep Neural Network Learning** We compared the performance of three different deep learning architectures to find an efficient method of detecting MDR for *Salmonella*, *Campylobacter*, and *Listeria*. In preliminary analyses, a generative adversarial network (GAN) proved more effective than Auto-Encoder for *Listeria* MDR, while a multi-layer perceptron (MLP) provided the best results for *Campylobacter* MDR. Additionally, *Salmonella* MDR classification was best performed with the Auto-Encoder design. **(a) Multi-Layer Perceptron:** An MLP [48] is a type of feed-forward artificial neural network that can distinguish data that cannot be linearly separated. These multi-layered networks consist of hidden nodes with a non-linear activation function that are connected with specific weights to the next layer of nodes. At the learning stage, connection weights are adjusted based on the amount of error in the output using a backpropagation function. **(b) Generative Adversarial Network:** A GAN [49] is a deep learning method for generating models from data using supervised learning techniques. Generative modeling involves discovering and understanding regularities and patterns in data. Rather than treating the problem as an unsupervised problem, GAN treats it as a supervised problem with two submodels: a generator model and a discriminator model. The generator model attempts to generate new samples from the negatives, while the discriminator model tries to determine what is positive and what is negative. Using backpropagation, we train the generator and discriminator models together. **(c) Auto-Encoder:** Autoencoder [50] is a stacked neural network layer system comprising an encoder layer, a latent or representative layer, a decoder layer, and an output layer. The latent layer will embed data without labels in a meaningful manner, and the output layer will attempt to recreate the original input. With the backpropagation algorithm, the networks are learned by minimizing the reconstruction error, which is the difference between the original and the reconstructed inputs.

For MDR classification using the above models, the prediction the confusion matrix, which is used to compare the model performance, is shown in Table 2. The ground truth has been presented as the actual value and the model’s prediction has been presented as the predicted value in this table. When the actual value and predicted value are both positive (+), this is known as a true-positive prediction in the confusion matrix (also known as the error matrix). False-positive predictions occur when the actual value is negative (−) and the predicted value is positive (+). A true-negative prediction is one in which both the actual and the predicted results are negative (−), while a false-negative prediction is one in which the actual is positive (+) and the prediction is negative (−). Precision is the measure of the proportion of correct positive predictions among the positive predictions. Similarly, recall measures the proportion of actual positives identified. Specificity refers to the number of correctly identified negative samples. Furthermore, the F1-Score is the harmonic mean of precision and recall. The purpose of these metrics is to evaluate the predictive ability of a model.

In order to avoid overfitting in our models, the scores are averaged from four-fold stratified cross-validation. The imbalanced dataset could be responsible for the high number of false negatives shown in the table for *Listeria* MDR (Table 2). Overall, our learning models are able to predict MDR with a greater than 86% F1-Score confidence. Considering the imbalance in the dataset, the prediction scores are reasonable (Table 2). Additionally, we generated receiver operating characteristic curves (ROC curves) to show the performance of our binary classification models at different thresholds.

**Figure 1:**
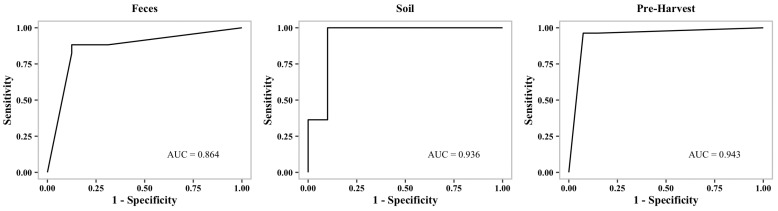
Receiver operating characteristic curve (ROC curve) of ***Salmonella*****MDR** models.

**Figure 2:**
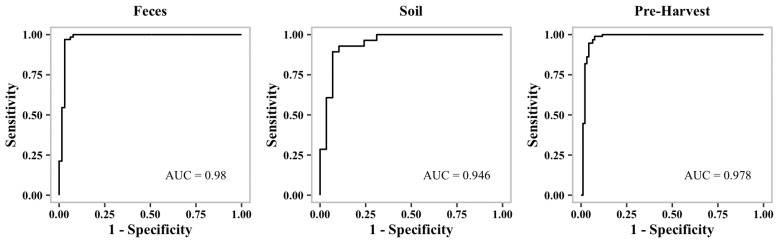
Receiver operating characteristic curve (ROC curve) of ***Campylobacter*****MDR** models.

**Figure 3:**
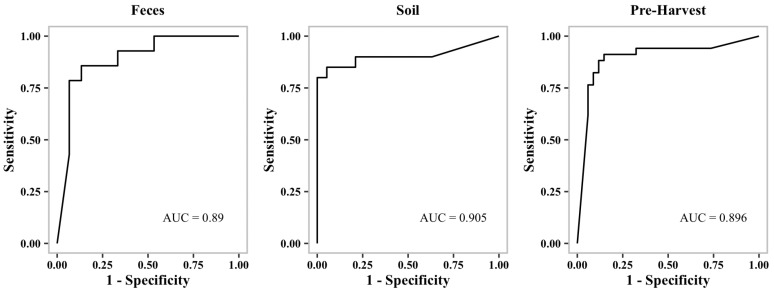
Receiver operating characteristic curve (ROC curve) of ***Listeria*****MDR** predictive models.

Figure 1, Figure 2 and Figure 3 show representative ROC curves of MDR models. AUC-ROC curves are generally used to measure the performance of classification problems at different threshold levels. It depicts the trade-off between the True-Positive Rate (sensitivity) and the False-Positive Rate (1—specificity). ROC represents a probability curve, while AUC represents the measure of separation. It is indicative of better performance when classifiers provide curves that are close to the top-left corner. According to the ROC plots, our MDR prediction models are able to distinguish the classes fairly well. For all MDR models, the AUC provides a distinguishing metric that is higher than 86%. It is an indication of the effectiveness of our classification models.

### 3.3. Critical Farm Feature Prediction

Our ensemble approach for selecting critical features utilized two traditional machine learning (RandomForest (RT) and eXtreme Gradient Boosting (XG)) and three deep learning (Multi-layer Perceptron (MLP), Generative Adversarial Network (GAN), and Auto-Encoder (ENC)) methods (see Section 2.5). In the RandomForest machine learning algorithm [51], individual decision trees that work together make up an ensemble of powerful algorithms. Using random samples from the dataset with replacement, random forest constructs several decision trees and predicts the outcome based on the majority vote. XGBoost [52] is another powerful decision-tree-based ensemble technique that incrementally improves the performance by adding new models in sequence to fix previous models’ errors. The gradient descent optimization algorithm minimizes the weak prediction loss of current models when adding new models.

The most influential variables agreed by at least three machine learning models and their rankings in individual algorithm predictions are enumerated in the following Table 3, Table 4 and Table 5. In machine learning, the approach determines the performance of the algorithm. There is no single model that is suitable for all applications. Thus, different models produce different results. To ensure reliability, majority voting is used in this study.

While Mg, P, and flock size appear to affect MDR in all pathogens, *Salmonella* and *Campylobacter* MDR is further affected by EC, Mn, flock age, and *Listeria*, and *Campylobacter* MDR appears to be affected by K, C:N, and Cu. *Salmonella* MDR alone is affected by pH, Na, Ca, water source, and housing type. *Campylobacter* MDR alone is affected by Zn, egg source, and brood feed and *Listeria* MDR by Cr and years of farming (Table 3, Table 4 and Table 5). However, as shown in Table 1 and reported earlier, the prevalence of *Campylobacter* MDR is negligible in the pastured poultry farms included in this study. Therefore, the focus will be on *Salmonella* and *Listeria* MDR in this manuscript.

### 3.4. Feature Values Associated with Low MDR Incidence

As described in Section 2.5, we use SHAP (SHapley Additive exPlanations) [26] to compute the effect of each feature on the model output. A model’s output with and without a specific feature is compared to determine its relative importance. When SHAP values are positive, they indicate greater importance than when SHAP values are negative. Feature values with negative SHAP values are recommended to lower the presence of multidrug resistance. We present our analysis of the most influential features in Table 6 and Table 7 along with value recommendations for reducing MDR.

SHAP dependency plots show the potential internal factors from feces that drive *Salmonella* MDR, such as EC and pH, while the external factors determined from the soil include P, Mn, and Na (Table 7). Only Mg appears to be critical for *Salmonella* MDR in both feces and soil. In *Listeria*, P, Cu, and Cr are correlated to MDR in feces while Mg, C:N, and years of farming are identified to be important for the MDR in soil (Table 6). Flock size and K appear to be critical for *Listeria* MDR in both soil and feces models. Although the exact mechanisms by which these farm management practices and physicochemical properties impact MDR are not fully understood, based on the current literature, we provide insights into the impact of variables on *Listeria* and *Salmonella* MDR.

#### 3.4.1. Drivers of *Listeria* MDR in Pastured Poultry

Possible mechanisms by which preharvest variables, specifically physicochemical properties of soil, contribute to the development of MDR are discussed below.

**Magnesium (Mg):** The Mg ion provides a strong cohesive force that strengthens bacterial ribosomes, the protein synthesis machinery. Its presence provides stability for the cell and counteracts the action of ribosome targeting antibiotics. Diminished levels of Mg have been shown to promote the activities of antibiotics and hamper the protein synthesis in bacteria [53]. The presence of Mg has also been reported to inhibit the transcription of genes involved in biofilm formation and promote the penetration of antibiotics [54]. In addition, Mg itself has been shown to have antimicrobial properties [55]. While ([56]) reported the Mg level of conventional poultry soil to be between 285 and 463 ppm, our analysis suggests that the optimum level of Mg to prevent MDR is <300 ppm. Pastured poultry farmers could utilize gypsum, a calcium sulphate salt that could reduce soil Mg by displacing the Mg within the soil with calcium.

**Phosphorus (P):** The buffering effect of phosphate formed from phosphorus in the bacterial culture medium has been reported to both enhance and diminish antimicrobial activities depending on the antimicrobial agents. Phosphate promotes the resistance of *S. lactis* to streptomycin but increases its sensitivity to tetracycline [57]. Moreover, the addition of 30 to 300 ug/L phosphorus has been shown to significantly increase biofilm formation in drinking water supply [58], which could potentially increase antibiotic resistance. Reducing the phosphorus level to below 5000 ppm as suggested by this study could potentially reduce MDR.

**Potassium (K):** Acesulfame potassium is an artificial sweetener found in various consumables and products such as soft drinks, jellies, beverages, and also poultry feed. The recent work of [59] shows that with increasing concentration of acesulfame potassium, and invariably potassium, the growth of bacteria with antibiotic resistance genes (ARGs) is inhibited. Contrarily, [60] show that uptake of K is essential for growth and antibiotic resistance in *Staphylococcus aureus*. While potassium levels in the conventional poultry litter and soil are reported to be between 3000 and 13,000 ppm, our models indicate that potassium levels between 7000 and 12,000 ppm are optimal to reduce the incidence of *Listeria* MDR. This supports the observation of [59] on the detrimental effects of potassium on antibiotic resistance bacteria.

**Carbon–Nitrogen Ratio (C:N):** Composting is one important way to remove undesirable antibiotics that are not metabolized by animals and that enter the environment through excretion. The C:N levels positively correlate and are indicative of the presence of ARGs in the environment [61]. Our predictive models recommend a C:N higher than 15 to mitigate MDR in *Listeria*. However, the recent work of [62] shows that a C:N of 26 is sufficient to remove ARGs from compost, and values higher than this may not be effective. Therefore, C:N values between 15 and 26 ppm are recommended to reduce MDR.

**Copper (Cu):** Based on Cu exposure studies with 96 microorganism isolates, Cu led to increased resistance to different clinically important antibiotics [63]. However, a comprehensive review on the effect of the antibiotic-binding capability of Cu indicates that it could both enhance or diminish antibiotic resistance [64]. This is not surprising, as Cu is an important cofactor for many enzymes required by bacteria. However, at elevated levels, it becomes toxic and acts as an antimicrobial and invokes adaptive response in the form of resistance from the organism [65]. Our prediction algorithms recommend Cu levels greater than 18 ppm to combat MDR.

**Chromium (Cr):** Investigation into the role of Cr in *Staphylococcus aureus* and *Escherichia coli* has established that it acts as an antibiotic and works synergistically with conventional antimicrobial agents to induce oxidative stress in the organisms [66]. Nevertheless, bacteria such as *Pseudomonas aeruginosa* have developed effective ways of reducing and ejecting chromium from the cell, thereby making it less potent. Increasing the level of chromium to > 3 ppm as predicted by our models could help prevent MDR in *Listeria*.

#### 3.4.2. Drivers of *Salmonella* MDR in Pastured Poultry

**Magnesium (Mg):** Similar to the observation with *Listeria*, Mg appears to be an important driver of MDR in *Salmonella*. Mg is important for stabilizing protein synthesis machinery. It is interesting to note that both *Salmonella* and *Listeria* exhibit MDR to classes of antimicrobials that target protein synthesis such as aminoglycosides, macrolides, glycylcycline, and ketolides. While the median range in conventional poultry for Mg is around 374 ppm, our prediction models recommend Mg levels lower than 300 ppm to destabilize bacterial ribosomes and increase the pathogen sensitivity to the antibiotics. Furthermore, the role of Mg in modulating nitrosative stress has been established [67]. Reduction in the Mg levels proposed in this study may lower *Salmonella* viability and increase its susceptibility to antimicrobial activity.

**Phosphorus (P):** P is capable of modulating buffering capacity of the pathogen environment as well as the capability to form biofilms, thereby potentially altering the sensitivity to antibiotics. Therefore, reducing access of *Salmonella* to phosphorus could reduce the MDR. Our model suggests P ≤150 ppm in the soil could help reduce *Salmonella* MDR. In an in vivo study, dietary and systemic increases in the level of phosphorus have been reported to lower pig mortality during *Salmonella* infection [68], possibly due to phosphorus-mediated stimulation of leukocyte production and defense mechanisms against the pathogen. Therefore, while P restriction in the poultry soil where *Salmonella* is found may be a good practice, supplementation of the poultry brood feed with P may enhance immune response and have a synergistic effect in further lowering presence and MDR of *Salmonella*.

**Electrical Conductivity (EC):** Treatment of MDR methicillin-resistant *Staphylococcus aureus* (MDR-MRSA) with chlorhexidine acetate nanoemulsion has been shown to have both in vivo and in vitro efficacy against the pathogen that correlated with an increase in the electrical conductivity [69]. ECs that range from 0.97 to 10.07 S/m, i.e., 970 mS/m to 10,070 mS/m, in graphene oxide have been reported to promote wound healing against MDR-MRSA. While EC levels detected in the samples in this study are below 1000 μS/cm, our models suggest that soil EC of 2000 μS/cm (200 mS/m) would be sufficient to reduce MDR incidence in *Salmonella*.

**pH:***Salmonella* is equipped with mechanisms to survive a wide range of pH between 4.4 and 9.0 with an optimum pH of around 7.0 [70]. Food safety regulation indicates acidic pH of 4.2 or lower is effective against *Salmonella* [71]. The recent work of [72] also indicates that a pH between 4.0 and 6.0 is sufficient to inhibit biofilm formation in *Salmonella*. The formation of biofilm is known to protect *Salmonella* from both in vitro and in vivo actions of antibiotics [73]. Our predictive model for soil suggests that a pH 6.5 could reduce MDR, possibly by inhibiting biofilm formation.

**Manganese (Mn):** Mn complex with antibiotic colistin has been reported to be effective against poultry avian pathogenic *Escherichia coli*, known for being highly antibiotic resistant. Mn in the metal complex form alone ([Mn(CO)${}_{3}(tpa-k^{3}N$)]Br) has antimicrobial activity, and synergistic combination with colistin further increases the killing efficiency of Mn [74]. The recommendation here to increase the Mn levels up to 70 ppm or above in the soil compared to 5 to 7 ppm found in the conventional poultry soil [56] could increase its availability and ultimately its antibacterial activity.

**Sodium (Na):** The presence of Na in the form of sodium chloride salt has been established to both increase the thermal and antibiotic resistance in multiple strains of *Salmonella* [75]. This was speculated to be a result of an increase in osmotic stress. Our predictive models suggest that decreasing the Na content of the soil to below 50 ppm could mitigate *Salmonella* MDR.

Factors such as water source, housing type, flock size, and age are identified as variables that could contribute to *Salmonella* MDR. However, the observational study described here does not account for factors that may influence such dynamic management practices. Future experiments that control for these changing variables are warranted.

## 4. Conclusions

In conclusion, while previous work established the presence of AR in pastured poultry with no historical use of antibiotics; in this study, we used machine learning and deep learning approaches to predict farm management practices and physicochemical properties of feces and soil that drive MDR in zoonotic poultry pathogens *Salmonella*, *Campylobacter*, and *Listeria*. Antibiotic use in agroecosystems is known to contribute to resistance. It is necessary to determine factors that contribute to the development of basal/background resistance in the absence of antibiotic selection pressure to understand its contribution in the animal–environment–human triad. Evaluation of the development of resistance in environments that are free of antibiotics, such as the all-natural antibiotic-free, pastured poultry production systems described here, are critical to understand the background AR. Understanding the drivers of background AR will aid future agroecosystem studies to determine the impact of antibiotic use in animal, environment and public health domains for designing optimal animal production systems to reduce AR in zoonotic pathogens, ensuring the safety of animal-based food products.

## Figures and Tables

**Table 1:** Antibiotic resistance profile of *Salmonella*, *Campylobacter*, and *Listeria* in soil and feces.

	Sample Type	Total No. of Samples with Bacteria	Number of Samples (Percentage) Resistant to		
			3 or More Antibiotics	1 or 2 Antibiotics	0 Antibiotics
*Salmonella*	Feces	114	31 (27%)	52 (46%)	31 (27%)
	Soil	83	31 (37%)	29 (35%)	23 (28%)
*Campylobacter*	Feces	343	16 (5%)	72 (21%)	255 (74%)
	Soil	150	8 (5%)	10 (7%)	132 (88%)
*Listeria*	Feces	101	73 (72%)	28 (28%)	0 (0%)
	Soil	114	97 (85%)	17 (15%)	0 (0%)

**Table 2:** The classification results of MDR predictive preharvest (feces and soil) models. Models are trained with farm practices and environmental physicochemical variables.

	Sample Type	Actual	Predicted		Precision	Recall	Specificity	F1-Score
			+	−				
*Salmonella*_MDR	Feces	+	16	1	0.88	0.94	0.83	0.9
		−	3	14				
	Soil	+	9	2	0.98	0.83	0.98	0.89
		−	0	10				
	Overall Preharvest	+	27	0	0.93	0.98	0.92	0.95
		−	2	25				
*Campylobacter*_MDR	Feces	+	65	0	0.94	1	0.94	0.97
		−	4	62				
	Soil	+	28	1	0.89	0.97	0.87	0.93
		−	4	25				
	Overall Preharvest	+	93	1	0.92	0.98	0.92	0.95
		−	8	86				
*Listeria*_MDR	Feces	+	12	3	0.94	0.80	0.95	0.86
		−	1	14				
	Soil	+	16	3	0.96	0.82	0.96	0.88
		−	1	19				
	Overall Preharvest	+	28	6	0.92	0.83	0.92	0.87
		−	3	31				

**Table 3:** The influential variables and their rank in respective algorithm for the prediction of *Salmonella* multidrug resistance (S_MDR). Please see Table A1 for variable descriptions. The ***top five*** ranks are shown in ***blue*** and the ***top ten*** in ***brown***. Overall, the ***top five*** most influential variables for *S_MDR* in feces are Mg, Water_Source_Well, Pasture_Housing_CTF, pH, and EC, while the ***top five*** for soil are Mg, P, Na, EC, and Ca.

SampleType	Model	Mg	P	K	Fe	EC	pH	Mn	Na	WaterSource Well	Pasture Housing_CT	Flock Size	Ca	Flock AgeDays	PastureHousing_CTF	BrSoy Free
**Feces**	**RT**	**1**				**5**				**8**	**6**			15		
	**XG**					14	**3**		**2**	**4**		**8**		**1**		
	**MLP**	**5**	**1**	**3**		**4**		**6**		**10**	**9**		**2**			
	**ENC**		**4**		**1**		**10**	**5**	**3**			**2**		**7**	11	
	**GAN**	**4**					**8**			**6**	**5**					
**Soil**	**RT**						**7**	**10**				**9**			**6**	**5**
	**XG**	**2**				**10**							**7**	**1**		
	**MLP**		**3**	**5**	**6**		**10**	**8**	**2**			**4**	**1**		12	
	**ENC**	**2**	**3**	**5**		**1**		**8**	**6**			**4**		**7**	**10**	
	**GAN**	**4**	**2**		**9**	**3**			**5**	**10**	12		**6**	**7**		

**Table 4:** The influential variables and their rank in their respective algorithm for the prediction of *Campylobacter* multidrug resistance (C_MDR). Please see Table A1 for variable descriptions. The ***top five*** ranks are shown in ***blue*** and the ***top ten*** in ***brown***. Overall, the ***top five*** most influential variables for *C_MDR* in feces are P, EC, Zn, Cu, and Mn, while the ***top five*** for soil are EC, K, FlockSize, Mg, and P.

SampleType	Model	P	EC	Mg	K	FlockSize	Mn	C:N	FlockAgeDays	Zn	Cu	Fe	Pb	EggSource	YearsFarming	BroodFeed
**Feces**	**RT**		**1**		13	**9**	**6**	**8**	**7**					**10**		
	**XG**									**3**	**1**			15		**7**
	**MLP**	**1**	**4**	**3**	**6**	**8**		**7**	**5**		**9**	**2**				
	**ENC**	**2**	**3**		**5**		**4**	**8**	**10**	**6**	**7**				**9**	14
	**GAN**	**4**	**6**	**3**	**5**		**10**		**7**	**8**		**2**			14	15
**Soil**	**RT**		**1**	**4**				**2**						13		
	**XG**		**3**	**4**	**5**							**2**		**6**		**7**
	**MLP**	**3**	**1**	**4**	**2**	**5**			**9**	**8**				11	14	
	**ENC**	**4**			**3**	**1**		**6**	**2**							
	**GAN**	**5**		**1**	**2**	**3**				**4**	**6**				**8**	

**Table 5:** The influential variables and their rank in their respective algorithm for the prediction of *Listeria* multidrug resistance (L_MDR). Please see Table A1 for variable descriptions. The ***top five*** ranks are shown in ***blue*** and the ***top ten*** in ***brown***. Overall, ***top five*** most influential variables for *L_MDR* in feces are K, P, FlockSize, Cu, and Cr, while the ***top five*** for soil are K, FlockSize, Mg, C:N, and YearsFarming.

SampleType	Model	K	FlockSize	P	Mg	EC	AvgNumBirds	Cr	Cu	C:N	Na	Cd	YearsFarming
**Feces**	**RT**	**1**		**3**	**7**	**6**		**8**	**4**	**10**	**5**		**2**
	**XG**		**5**									15	
	**MLP**	**1**					**2**	**10**	**6**		**5**		
	**ENC**	**3**	**5**	**2**			**4**			**8**		15	
	**GAN**	**3**	**2**	**1**		**6**		**9**	**5**				**8**
**Soil**	**RT**			**5**				**7**	**2**	**8**	**10**	11	15
	**XG**												
	**MLP**	**3**	**1**		**4**					**5**			
	**ENC**	**2**	**5**		**4**		**3**						12
	**GAN**	**2**	**1**		**5**				**8**	**7**	**4**		**10**

**Table 6:** **Pre-harvest** feature value recommendations for reducing for *Listeria* MDR Incidence.

Farm Variable	*Listeria* MDR	
	Feces	Soil
Mg		≤300
P	≤5000	
FlockSize	≮200	≯700
K	7000–12,000	>200
C:N		>15
Cu	>18	
Cr	>3	
YearsFarming		6–15

**Table 7:** **Pre-harvest** feature value recommendations for reducing *Salmonella* multidrug resistance.

Farm Variable	*Salmonella* MDR	
	Feces	Soil
Mg	≥2000	≤300
P		≤150
EC	≤2000	
pH	≤6.5	
Mn		≥70
Na		<50

## Data Availability

Restrictions apply to the availability of these data. Data were obtained from the Agricultural Research Service, USDA, and are available from Rothrock with the permission of the USDA.

## Abbreviations

The following abbreviations are used in this manuscript:

AR	Antibiotic resistance
MDR	Multidrug resistance
CDC	Centers for Disease Control and Prevention
RT	Random Forest
XG	eXtreme Gradient Boosting (XGBoost)
MLP	Multi-layer Perceptron
GAN	Generative Adversarial Network
ENC	Auto-Encoder
SHAP	SHapley Additive exPlanations
SMOTE	Synthetic Minority Oversampling Technique
S_MDR	*Salmonella* multidrug resistance
C_MDR	*Campylobacter* multidrug resistance
L_MDR	*Listeria* multidrug resistance
MDPI	Multidisciplinary Digital Publishing Institute
Mg	Magnesium
P	Phosphorus
K	Potassium
Fe	Iron
EC	Electrical Conductivity
pH	Potential of hydrogen
Mn	Manganese
Na	Sodium
Ca	Calcium
C:N	Carbon–Nitrogen Ratio
Zn	Zinc
Cu	Copper
Pb	Lead
Cr	Chromium
Cd	Cadmium

## Appendix A

**Table A1:** Poultry management practices.

Variable	Description
AvgNumBirds	Average number of birds that the farm handle in 1 year
AvgNumFlocks	Average number of flocks that the farm handle in 1 year
YearsFarming	Number of years the farm had been operating at the time of sampling
EggSource	Source of broiler eggs
BroodBedding	Type of bedding broilers received during brooding
BroodFeed	Up to top 3 sources of protein in brooding feed
BrGMOFree	Was the brood feed GMO free?
BrSoyFree	Was the brood soy free?
BrMedicated	Was the brood feed medicated?
BroodCleanFrequency	How often the brooding area was cleaned?
AveAgeToPasture	Average age broilers were put on pasture
PastureHousing	Type of pasture housing environment (CT: Chicken Tractor and CTF: Chicken Tractor with Fencing)
FreqHousingMove	How often the pasture area was moved?
AlwaysNewPasture	Was the pasture always moved to a brand-new pasture area?
PasturedFeed	Up to top 3 sources of protein in pasture feed
PaGMOFree	Was the pasture feed GMO free?
PaSoyFree	Was the pasture feed soy free?
PaMedicated	Were broilers medicated while on pasture?
LayersOnFarm	Were layers present on the farm?
CattleOnFarm	Were cattle present on the farm?
SwinOnFarm	Were swine present on the farm?
GoatsOnFarm	Were goats present on the farm?
SheepOnFarm	Were sheep present on the farm?
WaterSource	Water source for broilers during grow-out
FreqBirdHandling	How often chickens were handled on pasture?
AnyABXUse	Were antibiotics ever used on the broilers?
LengthFeedRestrixProcess	Length of feed restriction before processing
Seasons	Season of sample collection
FlockAgeDays	Age of flock at sample collection
Breed	Breed of broilers
FlockSize	Number of birds in the sample flock
